# Sugar, we’re going down: PRC2-mediated epigenetic repression of sucrose metabolism promotes phototrophy during seedling establishment

**DOI:** 10.1093/plcell/koaf155

**Published:** 2025-06-12

**Authors:** Rory Osborne

**Affiliations:** Assistant Features Editor, the Plant Cell, American Society of Plant Biologists; School of Biosciences, University of Birmingham, Birmingham B15 2TT, UK

In angiosperms, the plant life cycle consists of 3 major developmental phases: germination, vegetative growth, and reproduction. The transitions between these stages of sporophyte development are tightly regulated to ensure reproductive success. One such regulatory mechanism includes the stable downregulation of specific genes through the deposition of a repressive histone modification mark, H3K27me3. This modification promotes compaction of nucleosomes into transcriptionally inactive chromatin. By repressing the expression of genes required for the *previous* phase, plants have evolved to coordinate developmental transitions with specific environmental cues.

Arguably, the best-documented example of this mechanism is the repression of *FLOWERING LOCUS C* (*FLC*; a repressor of flowering) by the Polycomb Repressive Complex 2 (PRC2) during the winter to facilitate flowering in spring (De Lucia et al. 2008). In addition to *FLC*, PRC2 targets hundreds of other genes during each major phase transition of plant development, but the “what, where, and when” of these genes remains unclear (Bouyer et al. 2011). **Naseem Samo and coauthors (Samo et al. 2025)** now beautifully describe, in high resolution, the activity of PRC2 in both shoots and roots during the seed-to-seedling transition, where it targets embryogenesis and metabolism genes to support seedling establishment and photoautotrophy.

In their new work, the authors first identified the point during seedling establishment when primary metabolism switches from heterotrophy, characterized by the catabolism of seed storage molecules, to phototrophy, when atmospheric CO_2_ is taken up and converted into organic matter through photosynthesis. Determining that this transition occurs between 3 and 7 days after germination (dag), they next sought to describe both gene expression patterns and H3K27me3 dynamics during this metabolic switch. The authors performed mRNA and H3K27me3 chromatin immunoprecipitation (ChIP) sequencing on root and shoot tissues, both with and without PSII inhibition, a process that forces seedlings to remain heterotrophic. While these experiments identified several hundred putative PRC2 target genes, the most striking finding was that blocking PSII led to a considerable reduction in H3K27me3 deposition at the 7-dag stage. This suggested that epigenetic silencing at this time point is dependent on photosynthesis.

Supported by this observation, the authors hypothesized that PRC2 might participate in the transition to phototrophy through photosynthesis-dependent silencing. To test this, they studied an Arabidopsis mutant (hereby *cs*) lacking the PRC2 histone methyltransferase genes, *CURLY LEAF* and *SWINGER*. *cs* seedlings were significantly impaired in their ability to fix atmospheric CO_2_, and, interestingly, accumulated triacylglycerols, the major fatty acid storage molecule in dormant seeds. To understand these observations, the authors next performed mRNA sequencing of hetero- and phototrophic *cs* seedlings. Remarkably, the transcriptome of phototrophic *cs* closely resembled that of heterotrophic wild-type (WT) seedlings, while gene expression patterns in heterotrophic *cs* were closer to those of dry or germinating seeds. Metabolome profiling of germinating *cs* seedlings further corroborated these results by showing that they shared signatures with developing embryos.

Based on their findings, the authors suggested that PRC2 represses metabolic pathways associated with directing sugar metabolism toward lipid storage during seedling establishment, thus promoting phototrophic growth. To conclude their study, they cross-correlated the transcriptome of *cs* with their original H3K27me3 ChIP data in wild-type seedlings. This allowed them to identify *ISOCITRATE LYASE* (*ICL*; Eastmond et al. 2000), an essential gene within the glyoxylate cycle, as a novel PRC2 target, which becomes silenced during the seed-seedling transition to facilitate vegetative growth.

Taken together, these extensive data demonstrate 2 distinct phases of PRC2-mediated gene silencing (see Figure). From hydration of dry seed (imbibition) to 3 dag, genes associated with embryo development and maturation are first silenced independent of photosynthesis. Between 3- and 7 dag follows the photosynthesis-dependent suppression of genes that stimulate heterotrophy and lipid storage in seedlings, which promotes phototrophy. Beyond this new understanding of PRC2 activity, Samo et al. (2025) have also made available a wealth of high-quality omics data, which will hugely benefit the community.

**Figure.**
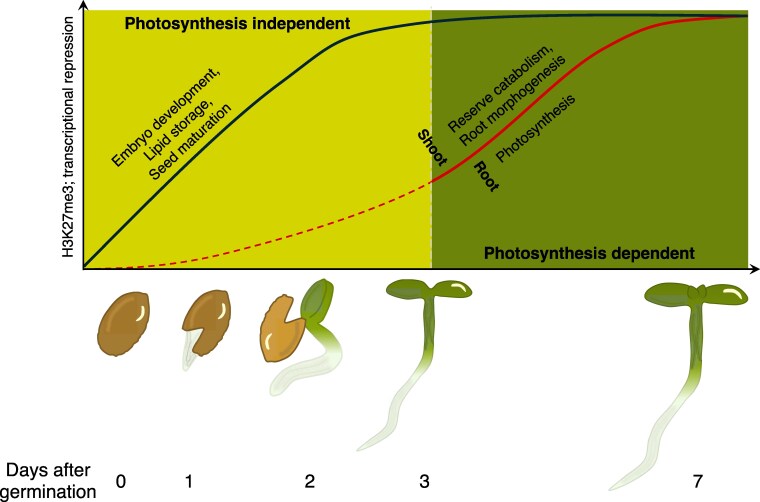
H3K27me3-mediated gene silencing dynamics during the seed-seedling transition. From zero to 3 dag, repression of genes that promote embryo development and seed maturation is photosynthesis independent. This is followed by the light- and tissue-dependent repression of genes associated with organ development and metabolism. Adapted from Samo et al. (2025), Figure 7.

## Recent related articles in *The Plant Cell*

- Wang et al. (2021) described the influence of rice SnRK1 kinase activity on the H3K27me3 demethylase JMJ705, which becomes activated under starvation conditions. This induces de-repression of key starvation genes, linking cellular energy status to chromatin structure and gene expression.
- Maugarny et al. (2024) showed that the effects of perturbations to PRC2 function on leaf morphology can be overcome through the simultaneous de-repression of *MICRORNA164B*, highlighting the robustness of biological systems.
- Pelayo et al. (2023) combined ChIP sequencing with mathematical modeling to identify novel biotimer genes repressed by PRC2 during flower development.

## Dive Curated Terms

The following phenotypic, genotypic, and functional terms are of significance to the work described in this paper:

FLC Gramene: AT5G10140
FLC Araport: AT5G10140

ICL Gramene: AT3G21720
ICL Araport: AT3G21720

JMJ705 Gramene: LOC_Os01g67970
JMJ705 Araport: LOC_Os01g67970
